# Effectiveness of Near Peer-Assisted Learning in Forensic Toxicology Teaching for Phase III MBBS Students: A Quasi-experimental, Pre-Test/Post-Test Study With Learner Perception Analysis

**DOI:** 10.7759/cureus.106409

**Published:** 2026-04-03

**Authors:** Anju Singh, Dinesh Kumar Singh, Ranvijay Singh, Vinay Tiwari, Amit Kumar Singh, Alok Kumar Arya, Snehanshu Shukla

**Affiliations:** 1 Department of Forensic Medicine, Rajarshi Dashrath Autonomous State Medical College (RDASMC), Ayodhya, IND; 2 Department of Transfusion Medicine, Rajarshi Dashrath Autonomous State Medical College (RDASMC), Ayodhya, IND; 3 Department of Forensic Medicine and Toxicology, Autonomous State Medical College (A.S.M.C), Hardoi, IND; 4 Department of Forensic Medicine and Toxicology, Rajarshi Dashrath Autonomous State Medical College (RDASMC), Ayodhya, IND; 5 Department of Microbiology, Rajarshi Dashrath Autonomous State Medical College (RDASMC), Ayodhya, IND

**Keywords:** academic performance, forensic toxicology, mbbs students, near peer-assisted learning, peer-assisted learning

## Abstract

Background and objective

Near peer-assisted learning (N-PAL) is an educational strategy where senior medical students teach junior students, and it is increasingly used to improve engagement, understanding, and retention in competency-based medical education. In concept-dense subjects such as forensic toxicology, reinforcement beyond lectures may be necessary to address passive learning and limited opportunities for clarification. In this study, we aimed to assess the effectiveness of N-PAL in improving academic performance among Phase III Part I Bachelor of Medicine and Bachelor of Surgery (MBBS) students and to evaluate students' perceptions of N-PAL as a teaching-learning method in forensic toxicology.

Methodology

A quasi-experimental pre-test and post-test study was conducted among 98 Phase III Part I MBBS students. Three forensic toxicology topics were selected and divided into subtopics. After faculty lectures, students were divided into small groups and taught through supervised N-PAL sessions by final-year MBBS students selected based on academic performance and willingness. Validated multiple-choice questions (MCQs)-based surprise pre-tests were conducted one week after lectures, and surprise post-tests were conducted one week after N-PAL sessions. Student perception was assessed using a 5-point Likert scale questionnaire.

Results

Post-test performance improved consistently after N-PAL sessions across all topics, showing a significant gain in learning. Students reported improved understanding, better retention, enhanced confidence, improved communication, and a comfortable learning environment. Most students expressed a preference for regular N-PAL sessions.

Conclusions

Supervised N-PAL is an effective reinforcement strategy in forensic toxicology, improving academic performance and student perceptions, while supporting confidence and communication within undergraduate medical education.

## Introduction

Didactic lectures delivered to large groups remain a commonly used teaching-learning strategy in undergraduate medical education, particularly in settings with limited faculty resources and extensive curricula [1]. These lectures enable uniform delivery of content and structured syllabus coverage within time-bound schedules [2]. However, lecture-based teaching promotes passive learning, with limited opportunities for interaction, discussion, and reinforcement [3]. These limitations are more pronounced in concept-dense subjects that require integration and interpretation [4]. Learners often struggle to retain information delivered in a single exposure, especially when the content has a high cognitive load and examination relevance [5].

Competency-based medical education emphasizes student-centered approaches that promote active participation, collaborative learning, and self-directed learning [6]. Curricula also require the development of competencies such as communication, teamwork, leadership, and confidence alongside academic knowledge [7]. Teaching strategies that facilitate interaction and peer engagement are therefore increasingly recognized as valuable supplements to traditional methods [8]. Peer-assisted learning (PAL) involves learners supporting each other’s academic development within a shared educational environment [9], either informally or through structured sessions integrated into the curriculum [10].

Near peer-assisted learning (N-PAL), where senior students teach juniors, is particularly relevant in medical education [11]. Senior students have recently undergone similar training and understand common misconceptions and learning challenges [12]. This cognitive proximity enables them to explain complex concepts in a manner that is aligned with the learners’ level of understanding [13]. Near-peer sessions are perceived as less intimidating than faculty-led teaching, encouraging active participation and question-asking [14]. This supportive environment reduces anxiety and enhances engagement during revision and reinforcement [15]. N-PAL therefore serves as an effective reinforcement strategy following lectures, particularly in subjects requiring deeper conceptual understanding [16]. Evidence suggests that structured and supervised peer-based teaching improves learner satisfaction and academic outcomes [17,18] and has been applied across both preclinical and clinical domains [19].

In preclinical education, PAL helps address challenges related to content volume and conceptual complexity [20]. In clinical settings, it supports the development of communication skills, examination techniques, and procedural competencies through interactive learning [1]. These approaches shift learning from passive listening to active discussion and problem-solving, enabling immediate clarification and feedback [2,3]. The approachability of near-peer tutors encourages learners to express uncertainties openly [4], contributing to improved understanding and retention [5]. PAL also benefits peer tutors by reinforcing knowledge through teaching, organization, and communication [6,7]. It fosters leadership, facilitation, and presentation skills essential in medical practice [8]. Early exposure to teaching roles aligns with professional expectations of educating peers, patients, and healthcare teams [9]. Near-peer teaching thus supports both academic and professional development within competency-based frameworks [10,11]. Student feedback consistently indicates that PAL is well received due to its interactive nature [12].

Despite increasing adoption, gaps remain in the structured implementation and evaluation of PAL in later phases of undergraduate medical education [13]. Most studies focus on preclinical years, with fewer addressing Phase III, where learning involves greater conceptual complexity and examination pressure [14]. Phase III Part I requires the integration and application of knowledge, making reinforcement strategies important [15]. Forensic Medicine and Toxicology is a concept-dense subject with clinical relevance, yet structured near-peer interventions in this domain remain limited [16]. Many studies emphasize learner satisfaction without consistently linking it to objective academic performance [17]. Institutions require measurable evidence of learning improvement to support curricular integration [18]. Multi-session pre-test and post-test designs provide stronger evidence but are less common in undergraduate settings. Implementation also requires faculty supervision to ensure conceptual accuracy and consistency, and evaluation of learner preferences is important for sustainable adoption.

The present study was conducted to evaluate the effectiveness of N-PAL in improving academic performance among Phase III Part I Bachelor of Medicine and Bachelor of Surgery (MBBS) students with respect to key competencies such as identification of toxicological agents, interpretation of forensic findings, and clinical correlation of toxicological concepts, and to assess student perceptions of its role as a teaching-learning strategy in undergraduate forensic toxicology education.

## Materials and methods

Study design and setting

The study was an interventional study conducted in the Department of Forensic Medicine and Toxicology at Rajarshi Dashrath Autonomous State Medical College (RDASMC), Ayodhya, India. N-PAL was considered the independent variable, while academic performance was the dependent variable. The study was designed to evaluate the effectiveness of N-PAL as a teaching-learning strategy following conventional didactic lectures. The intervention was implemented in a regulated educational setting under faculty supervision. Learner perception was also assessed using a structured questionnaire. The overall study framework was based on a pre-test and post-test comparison over three sessions.

Ethical approval and participant recruitment

The study commenced only after obtaining ethical clearance from the Institutional Ethics Committee, Rajarshi Dashrath Autonomous State Medical College (letter no. RDA SMC/IEC/2024/37). Participation was voluntary, and written informed consent was obtained from all participants. The inclusion criteria required Phase III Part I MBBS students who were willing to participate and provide consent, whereas students who were unwilling to participate were excluded. A total of 98 eligible students from the 2021-2022 MBBS batch were enrolled in the study. The anonymity of academic responses was maintained throughout the study. The intervention and assessments were conducted as part of scheduled academic reinforcement activities without disrupting the regular curriculum.

Selection of topics and development of learning material

Topics within the forensic toxicology curriculum were selected based on their complexity, examination relevance, and clinical importance. Three sessions were scheduled, each focusing on key themes essential for conceptual understanding and future clinical application. The selected topics were systematically divided into five subtopics to ensure a structured and organized delivery. Teaching content was developed by subject experts to maintain accuracy and alignment with curricular competencies. Assessment tools for each session included both multiple-choice questions (MCQs) and short-answer questions, which were validated before use. This structured approach ensured consistency in content delivery and assessment across all sessions.

Planning and structure of near peer-assisted learning sessions

Three N-PAL sessions were conducted following the corresponding faculty-led didactic lectures. The cohort of 98 students was divided into four groups of approximately equal size, designated as groups A, B, C, and D. Sessions were conducted in parallel batches, with one peer teacher assigned to each group. Peer teachers were final-year students selected based on academic performance and willingness to participate. Rotation of peer teachers across sessions provided broader exposure for senior students. Selected peer teachers underwent a structured orientation process before the sessions, which included a faculty-led briefing on session objectives, review of topic content, and guidance on effective teaching and group facilitation strategies. Tutors were required to prepare their assigned topics in advance, and the content was reviewed and validated by faculty members to ensure conceptual accuracy and uniformity across groups. The preparation period before each session allowed adequate familiarization with the material. Faculty members were oriented to the process and supervised the sessions to ensure content accuracy, consistency in delivery, and adherence to the planned structure.

Assessment strategy: pre-test and post-test

Assessment was conducted using 10 MCQs per session, which were prepared and validated in advance. Pre-tests were administered one week after the faculty lecture to assess baseline knowledge retention. Post-tests were conducted one week after the N-PAL sessions to evaluate reinforcement and consolidation of learning. The same group of 98 students participated in all three sessions. Scores were recorded and compared as pre-test and post-test results for each session. The assessment design accounted for both the difficulty level and the discrimination index of the questions. All assessments were administered through Google Forms to ensure standardized delivery and efficient data collection. The assessment tools, including multiple-choice and short-answer questions, were developed by the authors and were free from licensing restrictions.

Perception assessment tool

Student perception of N-PAL was assessed using a structured questionnaire based on a 5-point Likert scale [21], where 1 indicated strongly disagree, and 5 indicated strongly agree. The questionnaire evaluated multiple domains, including conceptual understanding, knowledge retention, comfort with the learning environment, communication skills, and confidence. The Likert scale is a non-proprietary and widely used measurement tool that does not require licensing or permission. The questionnaire was self-structured by the authors and did not involve any copyrighted or licensed instrument. Feedback was collected at the end of the sessions using Google Forms, and responses were tabulated. Perception outcomes were expressed as percentages of students selecting agreement categories, allowing a quantitative assessment of learner acceptability and the perceived educational value of N-PAL.

Data management and statistical analysis

Data were tabulated and analyzed using Microsoft Excel 2010 (Microsoft Corporation, Redmond, WA). Continuous variables were summarized using descriptive statistics, including mean and standard deviation (SD) for pre-test and post-test scores. Paired t-tests were applied to compare scores within each session, with statistical significance set at p < 0.05. Perception data were analyzed as percentages, with Likert responses grouped into broader categories. Survey findings were presented using bar charts for closed-ended questions. This analytical approach enabled evaluation of both academic performance and learner perceptions regarding N-PAL. All statistical methods used were standard, non-proprietary, and did not require licensing.

## Results

Academic performance: pre-test vs. post-test

Student performance was assessed using validated MCQ-based assessments conducted before and after the N-PAL sessions. The pre-test was administered one week after the faculty didactic lecture to evaluate baseline retention, while the post-test was conducted one week after the N-PAL intervention to assess reinforcement of learning. All 98 students who participated in the three sessions were included in a paired analysis of scores. Comparison of mean scores across sessions demonstrated a consistent improvement in post-test performance, indicating effective reinforcement of key concepts in forensic toxicology. Statistical analysis using the paired t-test showed that the observed improvement in scores was unlikely to be due to chance. The results for all three sessions (n = 98) are presented in Table 1, including mean scores with standard deviation, along with corresponding t-statistics, p-values, and critical t-values for one-tailed testing.

**Table 1:** Analysis of pre- and post-test of all three N-PAL sessions N-PAL: near peer-assisted learning

Metric	S1 Pre	S1 Post	S2 Pre	S2 Post	S3 Pre	S3 Post
Mean	3.72	7.93	4.60	8.43	3.39	7.01
Standard deviation	0.55	2.21	0.69	2.01	1.14	1.11
Observations	98.00	98.00	98.00	98.00	98.00	98.00
t-stat	48.26	37.15	20.03	—	—	—
P-value	0.00	0.00	0.00	—	—	—
t-critical (one-tailed)	1.66	1.66	1.66	—	—	—

**Figure 1:**
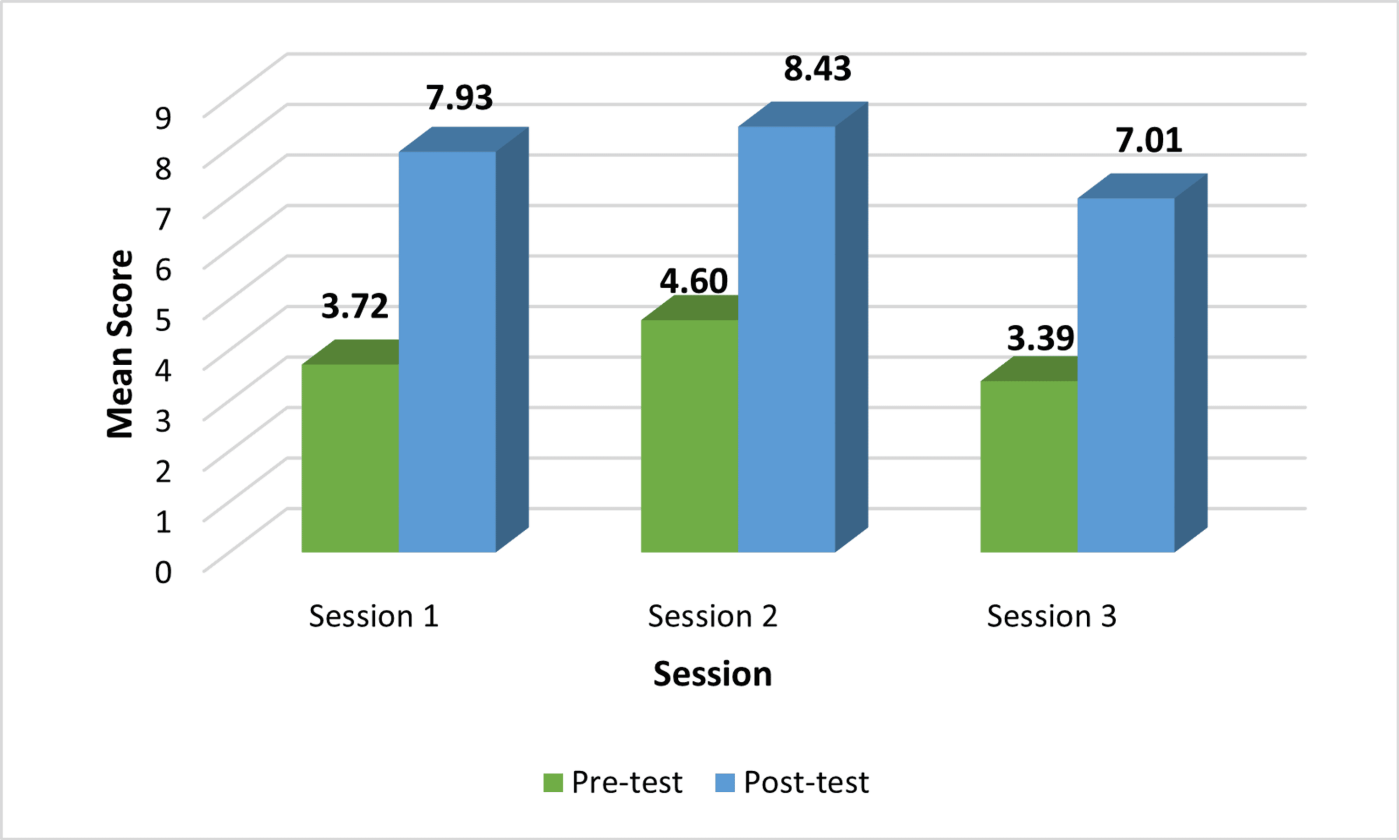
Pre-test and post-test score comparison across three N-PAL sessions N-PAL: near peer-assisted learning

Figure 1 shows the average scores before and after the test, in session 1, session 2, and session 3, which proves that there was a statistically significant difference in scores after the N-PAL.

Student perceptions of near peer-assisted learning

Student perception of N-PAL was assessed using a structured 5-point Likert scale questionnaire. Responses were analyzed by combining the categories of “Agree” and “Strongly Agree” to estimate overall positive perception. The questionnaire evaluated multiple domains, including conceptual understanding, knowledge retention, comfort within the learning environment, communication skills, and confidence. A clear majority of students reported positive responses across all evaluated domains, indicating that N-PAL was a well-accepted educational strategy. Among the assessed domains, the highest positive response was observed for confidence, followed by conceptual understanding and improvement in communication skills. These findings support the effectiveness of N-PAL as a learner-centered teaching approach for undergraduate students. The detailed distribution of student perceptions based on combined Likert scale responses (4 and 5) is presented in Table 2.

**Table 2:** Students' perception regarding N-PAL Data collected using a 5-point Likert scale [21] N-PAL: near peer-assisted learning

Sr. no.	Question	Students (n)	Percentage (%)
1	N-PAL session helps in conceptual understanding	78.00	79.59
2	N-PAL session helps in better learning and knowledge retention	69.00	70.40
3	N-PAL session makes the teaching environment more comfortable	68.00	69.38
4	N-PAL session helps in improving communication skills	74.00	75.51
5	N-PAL session helps in boosting confidence	83.00	84.69

Likert-scale response distribution for student perceptions

In addition to reporting overall positive perception as the combined "Agree" and "Strongly Agree" percentages, a detailed distribution of Likert-scale responses was analyzed for each perception item to provide a clearer understanding of response patterns, including neutral responses. Across all five items, no student selected “Strongly Disagree,” while only a minimal number of students selected “Disagree” for most statements. A noticeable proportion of students selected “Neutral,” particularly in domains related to knowledge retention and comfort within the learning environment, indicating that although most students perceived a benefit, some remained uncertain. The majority of responses were concentrated in the “Agree” category, followed by “Strongly Agree,” which reflects a broad acceptance of N-PAL as an educational strategy. The detailed distribution of student responses on the Likert scale for N-PAL (n = 98) is presented in Table 3.

**Table 3:** Likert-scale distribution of student perceptions of N-PAL sessions Data collected using a 5-point Likert scale [21] N-PAL: near peer-assisted learning

S. no.	Question	Strongly disagree	Disagree	Neutral	Agree	Strongly agree
1	N-PAL session helps in conceptual understanding	0.00	2.00	18.00	41.00	37.00
2	N-PAL session helps in better learning and knowledge retention	0.00	1.00	28.00	47.00	22.00
3	N-PAL session makes the teaching environment more comfortable	0.00	1.00	29.00	43.00	25.00
4	N-PAL session helps in improving communication skills	0.00	2.00	22.00	44.00	30.00
5	N-PAL session helps in boosting confidence	0.00	1.00	14.00	43.00	40.00

**Figure 2:**
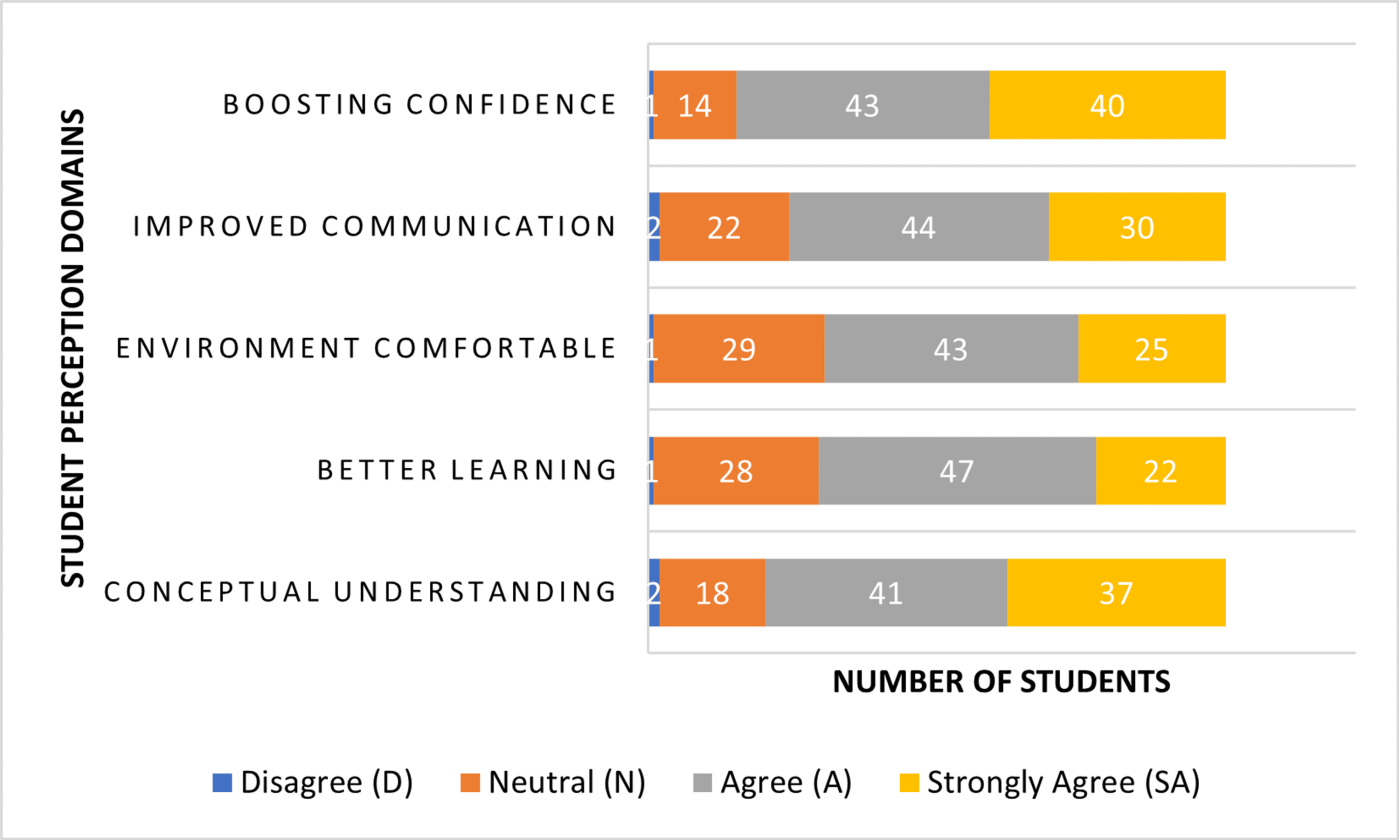
Student perceptions towards N-PAL (Likert-scale distribution) Data collected using a 5-point Likert scale [21] N-PAL: near peer-assisted learning

Figure 2 presents student responses across different perception domains, such as conceptual understanding, knowledge retention, comfort, communication skills, and confidence.

Preferred frequency of future near peer-assisted learning sessions

The acceptability of N-PAL sessions with respect to their future frequency was assessed based on student preferences, in order to evaluate the feasibility of integrating such sessions into the routine curriculum. A high level of acceptance for the continuation of N-PAL was observed, as no student expressed disinterest in future sessions. The majority of students preferred weekly sessions, suggesting that frequent reinforcement may be perceived as the most effective approach for consolidating complex concepts. A substantial proportion of students also preferred sessions to be conducted as needed, indicating a preference for flexibility depending on topic difficulty and examination relevance. Preferences for monthly and biweekly sessions were also reported, reflecting variability in individual learning needs. Overall, the distribution of preferences supports the continued implementation of N-PAL in undergraduate teaching. The detailed distribution of student preferences for future N-PAL session frequency is presented in Table 4.

**Table 4:** Desired frequency of N-PAL sessions N-PAL: near peer-assisted learning

Response option	Students (n)	Percentage (%)
Biweekly	15.00	15.30
Weekly	39.00	39.79
Monthly	20.00	20.40
Occasionally, as needed	24.00	24.49
Not Interested	0.00	0.00

## Discussion

This study evaluates N-PAL as a supportive educational strategy in teaching forensic toxicology to Phase III Part I MBBS students. The approach addresses limitations of traditional large-group lectures, particularly reduced opportunities for interaction, conceptual clarification, and long-term retention of complex content. Although lectures remain effective for delivering structured content, they promote passive learning and limit discussion and revision, especially in concept-dense subjects such as forensic toxicology. N-PAL was introduced as a learner-centred approach in which senior students facilitated learning in smaller groups [21]. This model is based on the premise that near-peer tutors, having recently acquired similar knowledge, better understand common learning challenges and misconceptions [17]. Such cognitive congruence enables simplified and relatable explanations, while social congruence fosters a supportive, less intimidating learning environment that encourages active participation [13].

In this study, N-PAL was implemented as a reinforcement strategy rather than a replacement for faculty-led teaching [22]. Students first received structured instruction through lectures, followed by N-PAL sessions designed to consolidate and reinforce learning [23]. This sequencing supports knowledge integration and retention through guided revision [24]. Faculty supervision ensured conceptual accuracy. Small-group formats enabled interaction, discussion, and clarification, shifting learning from passive reception to active engagement. In contrast to lecture-only approaches that rely predominantly on one-way information transmission, this blended model integrates reinforcement through interaction, thereby addressing limitations related to retention and learner engagement.

The effectiveness of N-PAL was assessed through objective academic outcomes and learner perceptions. Pre-test and post-test assessments demonstrated consistent improvement across all sessions, indicating enhanced recall and organization of knowledge. Compared with lecture-based teaching alone, where opportunities for iterative revision and feedback are limited, N-PAL provides structured reinforcement that supports consolidation of learning and reduces knowledge decay. Learner perception assessed through a Likert-scale questionnaire showed high acceptance and perceived benefit. Students reported improved conceptual understanding and greater comfort within the learning environment. The reduced hierarchical gap facilitated open communication and active participation. Compared with faculty-led tutorials or seminars, which may still retain hierarchical barriers, N-PAL offers a more approachable learning environment while maintaining academic structure through faculty oversight.

Students also reported improvements in confidence and communication skills, aligning with competency-based medical education objectives. While other active learning strategies, such as problem-based learning and team-based learning, emphasize analytical reasoning and collaborative problem-solving, they typically require substantial curricular restructuring, trained facilitators, and dedicated time allocation. Simulation-based learning further supports skill acquisition but is resource-intensive and infrastructure-dependent. In comparison, N-PAL functions as a low-resource, scalable intervention that can be integrated alongside existing lecture frameworks without a significant logistical burden.

From a pedagogical perspective, N-PAL occupies a distinct role among teaching modalities. Unlike lectures, it promotes active engagement; unlike problem-based or case-based learning, it is primarily focused on reinforcement rather than initial knowledge acquisition; and unlike simulation-based training, it does not depend on specialized infrastructure. This positions N-PAL as an effective adjunct strategy that complements rather than replaces other teaching methods, particularly in contexts requiring repeated revision of concept-dense material. The implications of these findings are relevant for curriculum planning in settings with large student cohorts and limited faculty resources. N-PAL provides a structured and sustainable method to enhance learning without increasing faculty workload. The approach appears particularly beneficial for topics with high cognitive load and examination relevance. Student preference for regular sessions further supports feasibility and acceptability, which are critical for long-term implementation.

The findings are consistent with literature demonstrating that peer-assisted learning improves academic performance and learner satisfaction [25]. Similar studies report enhanced engagement, conceptual clarity, and supportive learning environments [16], along with gains in confidence and communication skills [19]. However, some studies report improvements in satisfaction without corresponding objective gains [22]. In comparison with both traditional lecture-based teaching and other active learning strategies, the present study demonstrates that N-PAL achieves measurable academic improvement alongside positive learner perceptions, thereby establishing its relative pedagogical value as a structured, resource-efficient, and scalable reinforcement strategy in undergraduate forensic toxicology education.

Limitations and recommendations

This study was conducted within a single institution and involved a single cohort of Phase III Part I MBBS students, which may limit the generalizability of the findings to other educational settings with different curricula, teaching methods, or learning environments. The quasi-experimental design without randomization or a parallel control group limits the ability to establish a definitive causal relationship between the N-PAL intervention and the observed improvement in academic performance. The intervention was restricted to selected topics within forensic toxicology, and its effectiveness across the broader spectrum of forensic medicine remains to be determined. The study primarily assessed short-term academic improvement following reinforcement sessions, and long-term retention of knowledge was not evaluated. Although faculty supervision was maintained, variations in teaching style and communication among near-peer tutors may have influenced the learning experience across groups, introducing the possibility of tutor-related bias and variability in instructional quality. These factors should be considered when interpreting the findings and their applicability to wider educational contexts.

Future implementation of N-PAL could involve its integration as a structured and supervised reinforcement strategy across a wider range of topics within forensic medicine and toxicology, particularly those associated with great conceptual difficulty. Orientation or training programs for near-peer tutors may enhance consistency and effectiveness of teaching. Alignment of N-PAL sessions with lecture completion and examination schedules may further improve student engagement, confidence, and retention. Larger multi-institutional studies with extended follow-up periods are required to evaluate long-term retention, academic performance, and the broader applicability of N-PAL within undergraduate medical education.

## Conclusions

The study demonstrates that N-PAL is an effective reinforcement strategy for teaching forensic toxicology to Phase III Part I MBBS students, as its implementation under faculty supervision following traditional lectures creates an interactive and supportive learning environment that enhances conceptual understanding and improves retention of key concepts. The findings indicate that students not only showed improved academic performance after N-PAL sessions but also perceived benefits in terms of increased confidence, enhanced communication skills, and greater comfort in asking questions. The small-group structure, along with the cognitive and social proximity of near-peer tutors, facilitates active engagement and effective clarification of doubts, thereby addressing the limitations associated with large-group lecture-based teaching. In comparison to conventional lecture-only methods, N-PAL provides greater opportunities for interaction, feedback, and reinforcement of learning. While other active learning strategies, such as problem-based learning and team-based learning, focus on analytical reasoning and require greater structural and resource investment, N-PAL offers a comparatively simple, scalable, and resource-efficient approach that can be readily integrated within existing curricula. Student feedback further reflects strong acceptance of this approach, with a clear preference for its regular incorporation into the curriculum, supporting the integration of N-PAL as a structured pedagogical reinforcement strategy in forensic medicine education, particularly for conceptually demanding and high-yield topics that benefit from repeated engagement and interactive learning.
